# A Novel Semiautomatic Interpretation Model for Impulse Neutron Oxygen Activation Time Spectrum Data

**DOI:** 10.1155/2022/7395529

**Published:** 2022-10-10

**Authors:** Yong Dong, Mengxia Li, Ruiquan Liao

**Affiliations:** ^1^School of Information and Mathematics, Yangtze University, Jingzhou 434023, China; ^2^School of Computer Science, Yangtze University, Jingzhou 434023, China; ^3^Laboratory of Multiphase Pipe Flow, Gas Lift Innovation Center, CNPC, Yangtze University, Wuhan 430100, China; ^4^Petroleum Engineering College, Yangtze University, Wuhan 430100, China

## Abstract

The existing interpretation models for the time spectrum of impulse neutron oxygen activation require interpreters to select the peak range or background range manually from the time spectrum curve, and there is no adaptive interpretation model that can determine the peak range or background range. In this paper, an adaptive selection rule for background segment is proposed, and a semiautomatic interpretation model is constructed by combining background segment interpretation model. Firstly, the interpretation operator selects the time spectrum curve, then the algorithm program adaptively determines the background segment according to the rules, and then calculates and displays the transit time and volume flow according to the background segment interpretation model. The processing results of the measured data show that the interpretation model in this paper not only retains the interpretation precision of the background interpretation model, but also reduces the labor intensity of the interpretation operator, realizing the semiautomatic interpretation of the time spectrum.

## 1. Introduction

Impulse neutron oxygen activation logging is suitable for monitoring the flow direction and velocity of fluid containing oxygen atoms, which is not affected by formation porosity, fluid salinity, viscosity, and other factors. The tested space includes the space inside the tubing, the annulus space between the tubing and the casing, and the channeling space outside the casing. Moreover, the flow measurement has a wide range and has been widely used in many oilfields, especially in the monitoring of oil production by water and gas flooding. The literature [1–4] introduced the improvement of the design of the impulse neutron oxygen activation instrument. The literature [5] makes further analysis and mining of time spectrum data. The literature [6] introduced the methods to improve the efficiency of oxygen activation logging. The literature [7, 8] introduced the application of the oxygen activation logging method in the low permeability oil field and tight reservoir. A successful example of optimizing oil well production by combining impulse neutron oxygen activation with integrated production data analysis is presented in the literature [9]. The literature [10] used the method of function fitting to fit the whole time spectrum curve to extract velocity information. The literature [11] carried out a numerical simulation of the impulse neutron activation method. The literature [12] studied the feasibility of improving the interpretation accuracy of impulse neutron activation time spectrum.

In impulse neutron oxygen activation logging, the key to interpret fluid velocity is to accurately extract the transit time from the activation time spectrum, that is, the time it takes for the activation fluid to travel from neutron source to detector. The activation time spectrum reflects strong statistical fluctuation, which makes it difficult to accurately calculate the transit time. Therefore, researchers have established a variety of transit time interpretation models, mainly including three: traditional weighted average model [13, 14], peak function fitting model [15, 16], and background segment model [17].

Because the pulse seed oxygen activation logging instrument can obtain multiple time spectrum curve data in one measurement, the first step of all interpretation processes is to select a time spectrum curve for subsequent interpretation model invocation.

The peak function fitting interpretation model [15, 16] requires interpreters to set appropriate peak shape function according to the specific shape of spectrum peaks. However, the actual spectrum peaks and peaks are diverse, lacking quantitative discrimination standards, and interpretation results are greatly influenced by interpreters.

The traditional weighted average interpretation model is also called the Kappa Peak model [13, 14], which requires interpreters to manually select spectrum peak segments to participate in the calculation. As there is no unified selection standard for spectrum peak segments, interpretation results depend on interpreters' personal experience [17].

The background segment interpretation model [17] requires interpreters to select background segments, and its workload is equivalent to that of the traditional weighted average interpretation model. However, literature [17] shows that this interpretation model is little influenced by interpreters.

Existing interpretation models require interpreters to participate in specific interpretation processes, or set interpretation parameters, or select peaks, or select background sections. After a brief analysis of the existing interpretation models, this paper proposes a semiautomatic algorithm to select the background segment and constructs a new semiautomatic interpretation model based on the background segment model. Finally, the effectiveness of the new model is verified by the measured data, and the conclusion is given.

## 2. Introduction of Existing Time Spectrum Interpretation Models

**Figure 1:**
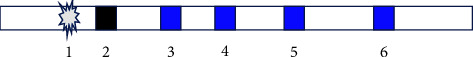
The structure diagram of the instrument (Meaning of codes, 1. Impulse neutron source; 2. Shield; 3. D4 probes; 4. D3 probes; 5. D2 probes; 6. D1 probes).

The structure diagram of the impulse neutron oxygen activation instrument is shown in Figure 1. The oxygenated fluid (such as water) flows from the left side to the right side along the outside of the instrument. The neutron source bursts will activate some of the oxygen atoms in the fluid. The activated oxygen atoms will decay and emit gamma rays, which are received by the probe. The instrument records the gamma ray intensity data at a certain time interval, which is called the time spectrum data, and the corresponding curve is called the time spectrum curve.

**Figure 2:**
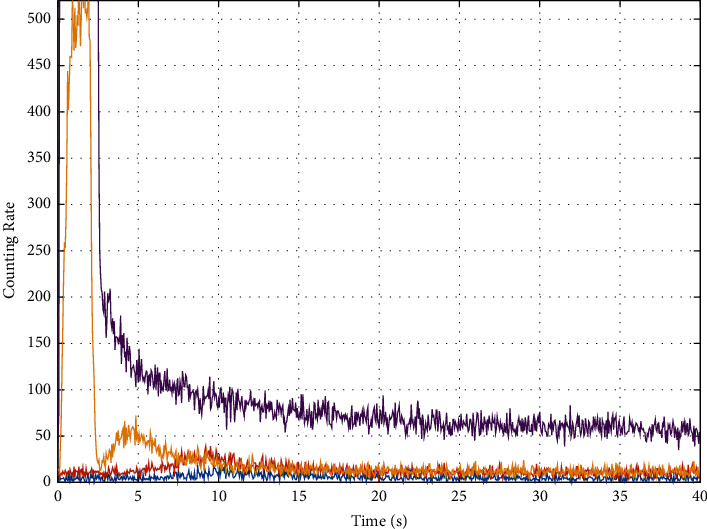
Time spectrum measured on well site.

The measured time spectrum is shown in Figure 2.

**Figure 3:**
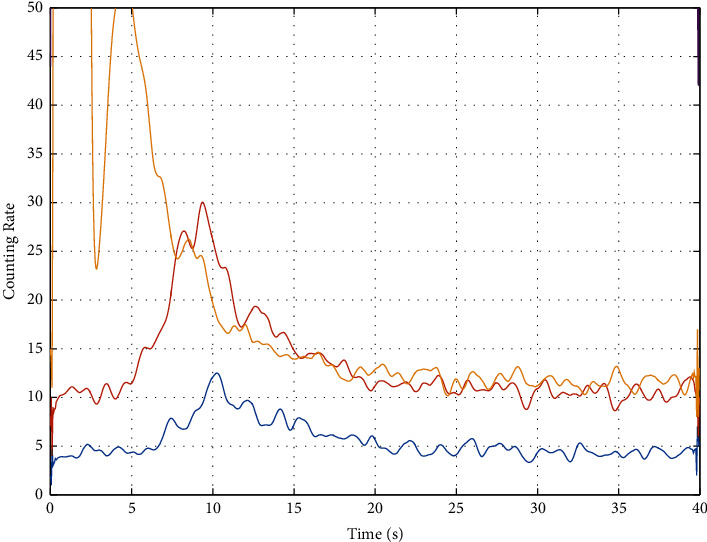
The results after 3 times of 9 points mean filtering method.

The time spectrum data in Figure 2 comes from the field measured data. It shows the time spectrum data recorded by D1 probes (blue), D2 probes (orange), D3 probes (yellow), and D4 probes (purple) in the impulse neutron oxygen activation instrument. The abscissa represents the time in seconds. The ordinate represents the count rate, which indicates the strength of the received signal. The value of the count rate reflects the relative strength of the signal at each moment in a measurement period without units. The statistical fluctuation of this time spectrum is very strong, which is not conducive to subsequent interpretation. Generally, a multipoint moving average method is adopted for filtering. In this paper, a 9-point average filtering method is adopted for three times filtering. The method takes out 9 data successively and calculates the arithmetic mean of these 9 data as the value of the fifth data. The filtering effect of the time spectrum in Figure 2 is shown in Figure 3. (Due to the difference in the ordinate display range, the data corresponding to the D4 probe in Figure 2 is not displayed in Figure 3).

### 2.1. Peak Function Fitting Model

Optional peak shape functions include Gaussian function, logarithmic Gaussian function [13], gamma function [13, 14], etc.

**Figure 4:**
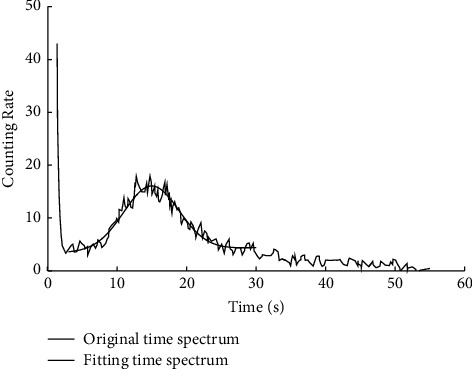
Fitting results of peak function.

The Figure 4 is the fitting result of the Gaussian function selected for a symmetric spectral peak in literature [14].

In the interpretation model, the symmetry of the spectrum peak needs to be recognized and judged by the interpreter with naked eyes, then the peak shape function is set, and the fitting is realized by the program, and the transit time is calculated according to the fitting parameters.

### 2.2. Weighted Average Model

The weighted average model is shown in the following equation [14]:

(1) $$\begin{array}{c} t_{m}=\frac{\sum_{i=T_{1}}^{T_{2}}\left(y_{i}t_{i}\right)}{\sum_{i=T_{1}}^{T_{2}}y_{i}}-\frac{1}{2}t_{b}\text{,} \end{array}$$

where *t*_*m*_ is transit time, *s*. *T*_1_ is the start time of the selected peak segment, *s*. *T*_2_ is the end time of the selected peak segment, *s*. *t*_*i*_ is the counting time between the start time and end time of the selected peak segment, *s*; *y*_*i*_ is the count rate corresponding to time *t*_*i*_. *t*_*b*_ is the length of time for the neutron to explode.

**Figure 5:**
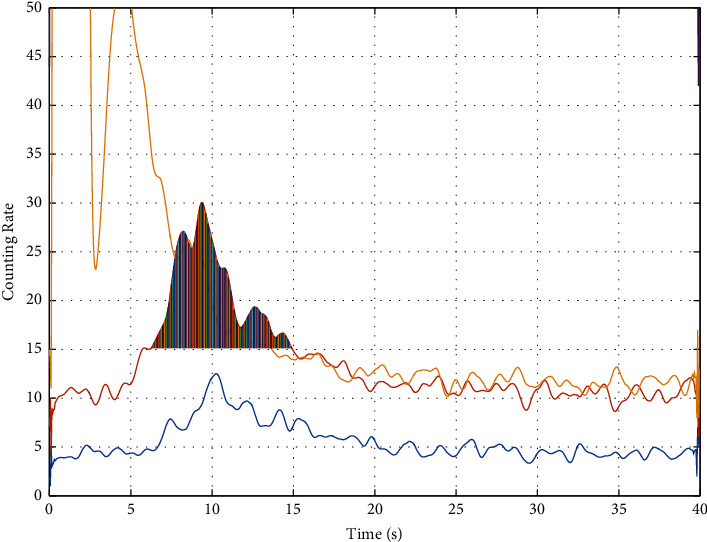
Peak selection schematic diagram of the traditional weighted average model.

As shown in Figure 5, the peak starting position needs to be manually selected. After selecting the starting position, the program automatically determines the termination position of the peak segment.

### 2.3. Background Segment Model

**Figure 6:**
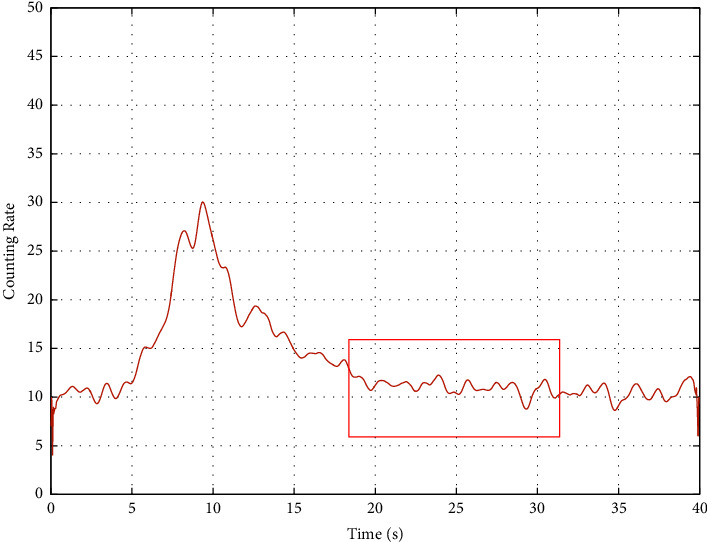
Schematic diagram of background section selection.

The background segment model comes from literature [17], and the results of comparative experiments show that this model is superior to the weighted average model. The background segment is the nearly horizontal segment in the time spectrum image, and its selection method is shown in Figure 6.

In literature [17], the interpreter identifies the horizontal segment manually and selects the background segment by dragging the mouse. The background segment interpretation model is different from the traditional weighted average model, which essentially increases the weight of points with a high count rate.

## 3. Semiautomatic Selection Method of Background Section

Literature [17] points out that the background segment is “the near-horizontal segment close to the spectral peak.” “Near horizontal refers to the fact that in the selected range, the count rate fluctuates around a horizontal line with basically the same fluctuation amplitude, and the overall change trend is like horizontal.

Figure 5 shows that the test period of time spectrum data is 40 seconds (neutron activation time is 2 seconds). As can be seen from Figure 5, for the filtered time spectrum curve, the spectrum peak should contain the maximum value of the spectrum curve, and the maximum value should not be within the neutron burst period (generally 0.8–2 seconds). Nor at the end of a test cycle (that is, near 40 in Figure 5); The approximate horizontal segment is located on the left or right side of the maximum value of the spectrum curve, and the duration of the approximate horizontal segment is more than 5 seconds, and the overall change trend of the approximate horizontal segment.

The original time spectral sequence is filtered by 9-point moving average for 3 times, and the subsequence with length *N* is selected, denoted as {*x*_*i*_}, *i*=1,2,…, *N*. The sequence is fitted by a linear function and the fitting equation is obtained.

(2) $$\begin{array}{c} y=ax+b\text{,} \end{array}$$

At the same time, the improved relative mean deviation of the subsequence is calculated.

(3) $$\begin{array}{c} r\;d=\frac{1}{N\times \max\;\left\{\left|\bar{x}\right|,1\right\}}\overset{N}{\underset{i=1}{\sum }}\left|x_{i}-\bar{x}\right|\text{,} \end{array}$$

where $\bar{x}=1/N\sum_{i=1}^{N}x_{i}$.

When the absolute value of the slope of the fitting line is small and RD is small, we can think that the subsequence is approximately horizontal. Combined with the calculation results of some data, the threshold recognition rules established in this paper are as follows:

(4) $$\begin{array}{c} \left|a\right|<0.1\text{,}\\ r\;d<0.15\text{.} \end{array}$$

Taking the time spectrum of the 40-second test period as an example, the automatic identification process of the near-horizontal segment is as follows:

1. For the time spectrum curve selected by the interpreter, the 9-point mean filtering is automatically performed three times, and the filtered data is denoted as *t*_*i*_, *i*=1,2,…, *N*, *N* indicates the data length.
2. Determine the time corresponding to the maximum value of the spectrum curve within the time range of 2 to 38 seconds, denoted as *t*_max_. If *t*_*m*_ > 20, we calculate parameters *a*_*i*_ and *rd*_*i*_ respectively for the time spectrum data in the following 5-second periods according to equations (2) and (3), the 5-second periods consist of [1+*i*, 6+*i*] second, *i*=1,2,…, 9. If *t*_*m*_ ≤ 20, we calculate parameters *a*_*i*_ and *rd*_*i*_, respectively, for the time spectrum data in the following 5-second periods according to equations (2) and (3), the 5-second periods consist of [24+*i*, 29+*i*] second, *i*=1,2, ⋯, 9.
3. Select a *a*_*i*_ and *rd*_*i*_ that meet the threshold rule, determine the corresponding time segment, and use the time spectrum data corresponding to the time segment as the near-horizontal segment.

## 4. Semiautomatic Interpretation Model of Time Spectrum and Its Verification

### 4.1. Semi-Automatic Interpretation Model

1. The time spectrum curve specified by the interpreter for interpretation.
2. Automatically determine the near-horizontal segment according to segment 2.
3. The near-horizontal segment determined in the previous step is taken as the background segment and interpreted according to the background segment model [17].

### 4.2. Verification of Semiautomatic Interpretation Model

The interpretation effect of the semiautomatic interpretation model is verified from two aspects. First, for the measured spectrum shown in Figure 1, the processing results of the background interpretation model and the model in this paper are compared. The second is to analyze the interpretation effect of the model based on the measured data of different Wells and different depths.

The volumetric flow interpretation model is as follows [17]:

(5) $$\begin{array}{c} Q_{V}=PC\times \frac{L}{t_{m}}\text{,} \end{array}$$

where *t*_*m*_ represents the transit time, *s*; *PC* represents the cross section area of the runner, m^2^; *L* is the source distance, *m*.

### 4.3. Comparison between Semiautomatic Model and Background Segment Model

Literature [17] has pointed out that the background segment model is superior to the traditional weighted average model and function fitting model.

As shown in Figure 2, after three times of 9-point moving average filtering, the spectral peak of the time spectrum corresponding to the D2 probe is obvious, and the near-horizontal segment is obvious. Therefore, THE TIME spectrum data of D2 is selected for interpretation.

The comparison of interpretation results between the automatic interpretation model and the background segment model in this paper is shown in Table 1.

For the semiautomatic interpretation model, repeat 10 times, the volumetric flow rate of interpretation remains unchanged.

It can be seen from the data in column 2 of Table 1 that the near-horizontal segment [26.00, 31.00] determined by the semiautomatic model is inside the near-horizontal segment manually selected in the background segment model, indicating the feasibility of the semiautomatic model.

The data in column 3 of Table 1 show that the interpretation result of the semiautomatic model is consistent with that of the background segment model.

### 4.4. Interpretation Effect of Repeated Measurement Data

In the actual logging process, some depth points will be measured repeatedly. The instrument depth remained fixed during the two measurements, and the measurements were taken only 1-2 minutes apart. Therefore, it can be considered that the volume flow rate of the fluid is approximately stable with little change in the repeated test process, and its corresponding time spectrum should be consistent to a certain extent, and the flow rate obtained by interpretation should also be approximately consistent. For repeated measurement data from different Wells and different depth measurement points, the measurement point information and explained flow are shown in Table 2.where the relative error is defined by the following equation:

(6) $$\begin{array}{c} Relative\;error=\frac{Maximum\;interpretive\;flow-Minimum\;interpretive\;flow}{Minimum\;interpretive\;flow}\text{.} \end{array}$$

As we can see from columns 1 and 2 of Table 2, the measurement points are in different Wells at different depths. Repeat the program five times and the result remains the same. As can be seen from columns 3 and 4 of Table 2, pressure and temperature recorded by repeated measurements are basically consistent at the same measurement point. As can be seen from column 5 of Table 2, the near-horizontal segment recognized by the semiautomatic model is not completely the same at the same measurement point, which reflects the randomness of the oxygen activation time spectrum signal. As can be seen from Columns 6 and 7 of Table 2, the flow interpreted based on repeated test data within a short period of time at the same measurement point has a high consistency, and its relative error is less than 2%. It shows that the semiautomatic interpretation model shows that the semiautomated model has high interpretation stability and accuracy.

## 5. Conclusion

1. Compared with the traditional weighted average interpretation model, function fitting model, and background segment interpretation model, the automatic interpretation model in this paper further reduces the workload of interpreters, it only needs the time spectrum curve selected by the interpreter for calculation, which is conducive to the design of fully automated interpretation model.
2. The automatic model in this paper further reduces the influence of the interpreter's experience on the interpretation results and has the same precision and interpretation stability as the background segment model.

## Figures and Tables

**Table 1:** Treatment effect of different model.

Model	Near-horizontal segment (*s*)	Volumetric flow rate (*m*^3^/*d*)
Background segment model	20.85–26.35	29.86
	25.75–31.85	29.84
	23.05–26.90	29.86
	23.55–37.15	29.86

Semiautomatic model	26.00–31.00	29.87

**Table 2:** Treatment effect of model in this paper.

Well number	Depth (m)	Pressure(MPa)	Temperature (°C)	Near horizontalsegment (s)	Volumetric flow rate(m^3^/d)	Relativeerror (%)
Well 1	175.5	9.01	−1.46	32–37	174.49	1.6
		9.0	−1.49	32–37	177.22	

Well 2	810.1	8.33	33.4	33–38	29.95	1.2
		8.33	33.4	33–38	29.60	

Well 3	2266.0	20.2	67	25–30	138.20	1.7
		20.2	67	28–33	140.60	
		20.2	67	27–32	138.60	

Well 4	2310.1	21.9	68.6	30–35	78.59	0.8
		21.9	68.6	28–33	79.21	

## Data Availability

The data used to support the findings of this study are available from the corresponding author upon request.
